# An optimized NGS sample preparation protocol for *in vitro* CRISPR screens

**DOI:** 10.1016/j.xpro.2021.100390

**Published:** 2021-03-15

**Authors:** Corrin A. Wohlhieter, Fathema Uddin, Àlvaro Quintanal-Villalonga, John T. Poirier, Triparna Sen, Charles M. Rudin

**Affiliations:** 1 Graduate Program in Pharmacology, Weill Cornell Medicine, New York, NY 10021, USA; 2 Department of Medicine, Memorial Sloan Kettering Cancer Center, New York, NY 10065, USA; 3 Perlmutter Cancer Center, New York University Langone Health, New York, NY 10016, USA; 4 Molecular Pharmacology Program, Memorial Sloan Kettering Cancer Center, New York, NY 10065, USA

**Keywords:** Cancer, High Throughput Screening, Molecular Biology, CRISPR

## Abstract

This standardized protocol describes the preparation of PCR amplified and purified samples from human cell lines passaged and collected from CRISPR screening. High-quality samples can be used to perform next-generation sequencing (NGS) to uncover changes in sgRNA abundance from the timepoint at which library-transduced cells are selected to the timepoint when the screen is ended. Here, we describe proper calculation methods for library representation and show how to overcome potential issues often encountered by researchers.

For complete information on the use and execution of this protocol, please refer to Wohlhieter et al. (2020).

## Highlights

• Calculate sgRNA representation to achieve adequate depth of coverage
• Amplify sequencing regions using optimized PCR primers
• Purify PCR samples to pass quality controls for next-generation sequencing
• Include proper controls to help troubleshoot issues with sample preparation

## Graphical abstract

**Figure:**
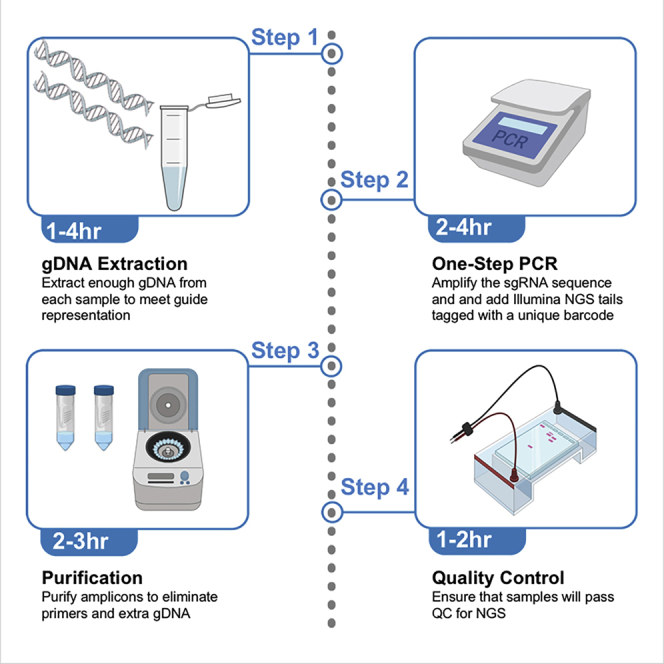

## Before you begin

This protocol assumes that the user has already performed a CRISPR-Cas9 screen *in vitro* and is prepared to collect cells to process for next-generation sequencing. As an additional resource, our lab has previously published a review article describing the design, execution, and analysis of pooled *in vitro* CRISPR-Cas9 screens (Miles et al., 2016).

**Figure 1:**
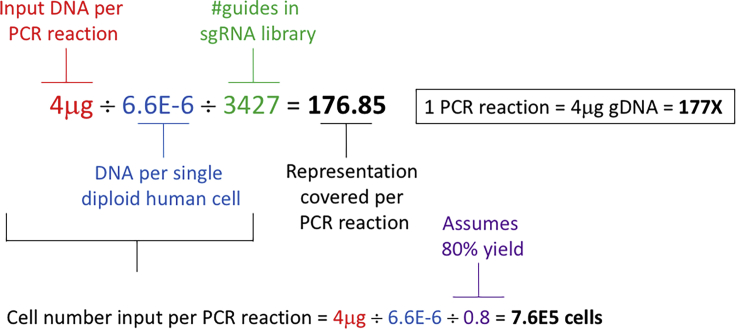
Example calculations for Saturn V library pool 1 This calculation is used to obtain the library representation present in one PCR sample. The color-coded variables describe the purpose of each value. Numbers in this example assume the use of human cell lines and Saturn V library pool 1 but can be adapted to other experimental conditions.

This protocol describes specific steps for preparation of samples for next-generation sequencing (NGS) from human cell lines transduced with a lentiviral sgRNA library. As an example for this protocol, we use the Saturn V CRISPR library containing the lentiGuide-PuroV2 backbone. The Saturn V library is designed as five independent pools (as outlined in Table 1) with each pool containing gene targets characterized by their “druggability” (Oprea et al., 2018). This protocol can be used for any library containing the lentiGuide-PuroV2 or lentiGuide-Puro backbone. Alternatively, this protocol can be adapted for use with other libraries by designing appropriate forward and reverse primers and calculating guide representation as outlined in Figure 1 to ensure that the library of sgRNAs are adequately covered. The genomic DNA (gDNA) yield represented in the calculations (Figure 1) was established and standardized by our laboratory in the human cell line A549 and would need to be established for use in a different model system. Most human cell lines have a similar yield to A549 cells; this protocol was successfully used for NCI-H358, NCI-H292, NCI-H82, NCI-H69, NCI-H526, and DMS114 cell lines.

**Table 1:** Library coverage for Saturn V pools

Saturn V Pool #	Number of guides	Library representation	Minimum no. cells for gDNA extraction	Total input genomic DNA required (μg)	Parallel PCR reactions (4 μg gDNA/reaction)
1	3,427	177X	760,000	4	1
		530X	2,300,000	12	3
		1061X	4,600,000	24	6
2	3,208	189X	760,000	4	1
		567X	2,300,000	12	3
		945X	3,800,000	20	5
3	3,184	190X	760,000	4	1
		571X	2,300,000	12	3
		952X	3,800,000	20	5
4	1,999	303X	760,000	4	1
		606X	1,500,000	8	2
		910X	2,300,000	12	3
5	2,168	280X	760,000	4	1
		559X	1,500,000	8	2
		1118X	3,000,000	16	4

The following table lists the library representation covered by one PCR reaction per pool and gives 2 additional examples for the number of PCR reactions required to cover a certain library representation. These values can be calculated as described in Figure 1 and can be adopted accordingly for any library. We recommend processing samples to cover a library representation of at least 300X or higher for high-quality NGS products (Miles et al., 2016).

### Decontaminate PCR workstation

PCR preparation should be performed inside a decontaminated PCR workstation to avoid cross-contamination between unique samples.

1. Spray the PCR workstation area with RNase AWAY, which eliminates DNA in addition to RNases, and thoroughly wipe.
2. Autoclave microcentrifuge and PCR tubes and pipette tips before placing them in the PCR workstation.
3. Place all needed microcentrifuge tubes and/or PCR tubes on racks inside the PCR workstation with the caps open.
4. Turn on UV light for at least 20 min to decontaminate workstation.
5. Turn off UV light and proceed to gDNA extraction.

***Note:*** We recommend performing gDNA extraction, PCR setup, and template addition in the decontaminated workstation using separate pipettes to avoid contamination.

## Key resources table

**Table:** 

REAGENT or RESOURCE	SOURCE	IDENTIFIER
**Critical commercial assays**		

PureLink Genomic DNA Mini Kit	Invitrogen	K1820-01
Qubit dsDNA BR Assay Kit	Invitrogen	Q32853
Qubit dsDNA HS Assay Kit	Invitrogen	Q32854
Qubit Assay Tubes	Invitrogen	32856
PCR tubes	Thermo Scientific	AB-0266
RNase *AWAY*	Molecular BioProducts	7002
Qubit dsDNA BR Assay Kit	Invitrogen	Q32850
GeneJET PCR Purification Kit	Thermo Scientific	K0702

**Chemicals, peptides, and recombinant proteins**		

NGS-adapted forward and reverse primers with barcodes	In house	N/A
Exonuclease I	New England Biolabs	M0293L
Herculase reagents	Agilent Technologies	600675
Saturn V Library (or other sgRNA lentiviral library)	In house	N/A

**Other**		

PCR workstation	AirClean Systems	AC624LFUV

## Materials and equipment

This protocol begins with the completion of cell passaging and collection from an *in vitro* CRISPR screen using human cell lines. Be sure to collect enough cells from each sample to meet the intended library representation (Figure 1). At least 16 cell doublings is recommended to ensure changes in sgRNA representation will be captured. These changes may show sgRNA depletion (negative selection screen) or sgRNA enrichment (positive selection screen). The number of doublings may change if the screen uses additional variables like pharmacological agents.

## Step-by-step method details

### gDNA extraction

**Timing: 1–4 h**

**Figure 2:**
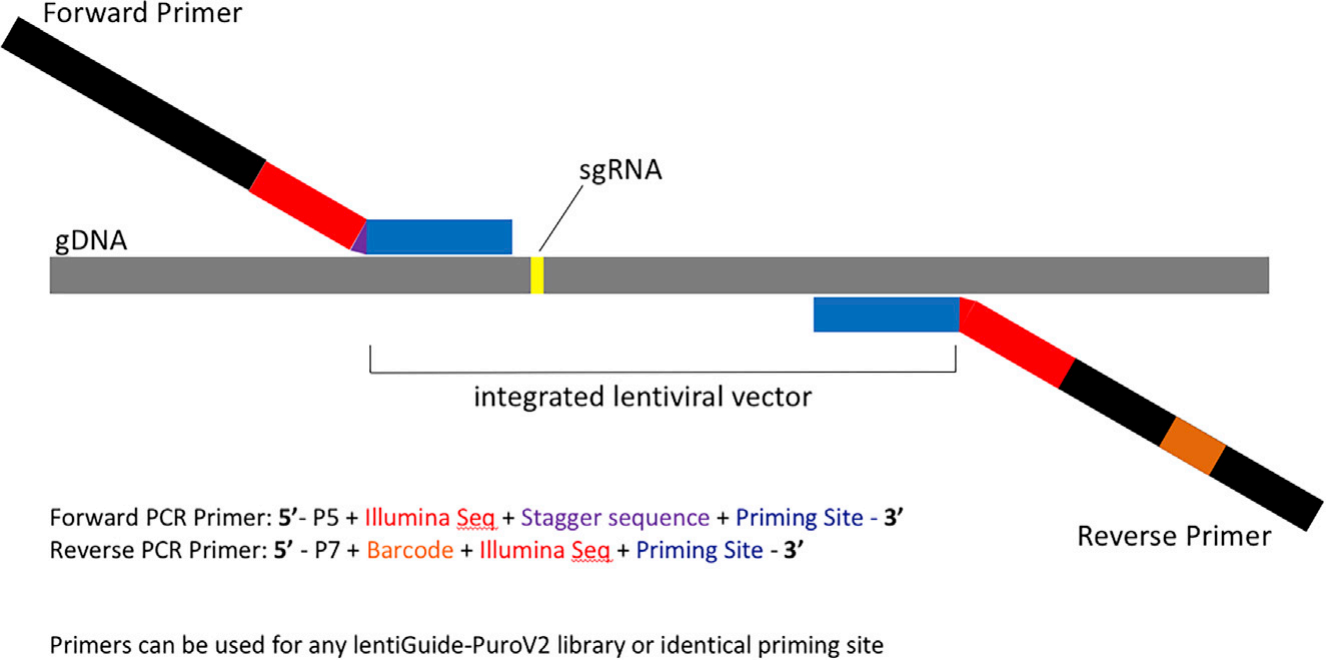
Forward and reverse primer design Forward PCR primers were designed to create a stagger sequence between the priming site and the Illumina sequence to facilitate diversity during NGS reads. Ten forward primers are pooled in each reaction with a stagger length of 1-10 bases. Reverse primers are designed to introduce a unique barcode to each sample. This barcode is used during analysis to identify individual samples within a pool. Details on primer design can be found in Tables 2 and 3.

The following procedure is used to harvest gDNA from cells that have undergone CRISPR screening. Cells that have been transduced with a lentiviral library have integrated genomic DNA containing an sgRNA sequence and adaptor sequences complimentary to the forward and reverse primers (Figure 2). The harvested gDNA will be used later for one-step PCR sample preparation for NGS. Refer to Table 1 to obtain the minimum number of cells required for gDNA extraction for your selected library coverage.

1. Harvest and centrifuge the selected number of cells (Table 1) in 1.5 mL microcentrifuge tubes at 300 × *g* for 3 min at 20°C. Do not pellet more than 5 million cells per microcentrifuge tube. Use an automated cell counter or equivalent to count your cells.

***Note:*** The maximum capacity for PureLink Genomic DNA spin columns is ∼5 million cells for gDNA extraction.

**Pause point:** You may store dry cell pellets at −80°C for up to 1 month or proceed to gDNA extraction.

2. Use PureLink Genomic DNA extraction kit following manufacturer’s protocol to extract gDNA from cell pellets. Elute in Molecular Grade Water in final step. Please refer to the manufacturer’s instructions for gDNA extraction using the following link: (http://tools.thermofisher.com/content/sfs/manuals/purelink_genomic_man.pdf)

**CRITICAL:** Do not process more than 5 million cells per column as this may cause the spin columns to clog and will decrease your yield. If more than 5 million cells is required to meet the depth of library coverage, extract gDNA in multiple spin columns and pool the gDNA after extraction.

**CRITICAL:** Ensure that there is no remaining volume of wash buffer above spin-column filters after centrifugation during washing steps. Repeat centrifuge step if there is a remaining volume of wash buffer above the spin column. Ethanol contamination from wash buffers will decrease your gDNA yield during the elution step.

***Note:*** Aim for a final concentration of at least 190 ng/μL (this is the minimum concentration needed to input 4 μg of gDNA into a single 50 μL PCR reaction). Generally, eluting in 50 μL Molecular Grade Water for 5 million processed cells yields concentrations >200 ng/μL.

***Optional:*** Perform a second elution with 20–30 μL additional Molecular Grade Water to recover more gDNA.

3. Obtain concentrations of extracted gDNA with Qubit dsDNA BR Assay Kit following manufacturer’s protocol (https://assets.thermofisher.com/TFS-Assets/LSG/manuals/Qubit_dsDNA_BR_Assay_UG.pdf)

**Pause point:** You may store gDNA samples at −20°C for the duration of your experiment, or proceed to one-step PCR sample preparation. Your gDNA samples can be stored at −20°C for over 10 years.

### One-step PCR sample preparation for NGS

**Timing: 2–4 h**

The following procedure is for one-step PCR NGS sample preparation. Forward primers are designed to include a priming site adjacent to the guide spacer sequence and introduces the P5 Illumina adapter, as well as stagger sequences to allow for diversity during NGS reads. Reverse primers are designed to include a priming site adjacent to the UMI sequence and introduces the P7 Illumina adaptor as well as a unique barcode to allow for multiplexed NGS (Figure 2).

4. Thaw frozen PCR reagents.

***Note:*** Thaw 5× Herculase buffer and dNTP Mix on top of ice and keep on ice after they are thawed. All other PCR components (other than Herculase) can be thawed at 20°C.

***Note:*** Keep Herculase on ice or at −20°C until needed.

***Note:*** DMSO takes roughly 30–40 min to thaw at 20°C. You may aliquot DMSO into smaller volumes to save time thawing.

5. Mix equal volumes of each 10 μM forward primer 1–10 to make a pool of all 10 forward primers and use this during PCR setup.

***Note:*** Each of the forward primers is one base shorter than the next (Tables 2 and 3). The different length primers improves NGS reads by adding diversity to the reads.

6. Set Up PCR master mixes and negative control reactions for each sample (Table 4).

**Table 4:** PCR setup

PCR component	Single PCR reaction	Master Mix example (10 parallel PCR reactions)	Master Mix example for negative control (10 PCR reactions)
gDNA	4 μg	40 μg	-
5× Herculase Buffer	10 μL	100 μL	100 μL
2.5 mM dNTP Mix	4 μL	40 μL	40 μL
Pooled Forward Primers (10 μM)	4.5 μL	45 μL	45 μL
Unique Reverse Primer (10 μM)	4.5 μL	45 μL	-
DMSO	3.75 μL	37.5 μL	37.5 μL
Herculase	2 μL	20 μL	20 μL
Molecular Grade Water	Bring volume up to 50 μL	Bring volume up to 500 μL	212.5 μL

For the negative control master mix, unique reverse primers and template gDNA are not included. Instead, reverse primers are added to each reaction separately for each unique reverse primer used for the negative control PCR reactions. gDNA template is not included in the negative control reactions to ensure that PCR components are not contaminated.

***Note:*** There should be a negative control for every reverse primer used (i.e., A PCR setup for 10 samples with 10 unique reverse primers should also have 10 negative controls, with at least one negative control for each unique reverse primer). gDNA template is not included in the negative control reactions to ensure that PCR components are not contaminated.

7. Vortex master mixes for 15–20 s and spin down.
8. Aliquot 50 μL of the master mix to respectively labeled tubes.
  a. For negative controls, aliquot 45.5 μL of negative control master mix and add 4.5 μL of 10 μM unique reverse primer to respectively labeled tubes and vortex.
  b. Spin down tubes at maximum speed for 10 s to ensure that all the liquid is at the bottom of the tube.
9. Proceed to thermocycling (see Table 5). The Denature, annealing, and extension steps should occur in order for 24 total cycles.

**Table 5:** Thermocycling conditions for one-step PCR

Steps	Temperature	Time	Cycles
Initial denaturation	98°C	3 min	1
Denaturation	98°C	30 s	24
Annealing	52°C	30 s	24
Extension	72°C	45 s	24
Final extension	72°C	10 min	1
Hold	4°C	infinite
10. Pool parallel PCR reactions and vortex to thoroughly mix (i.e., pool all PCR reactions with Sample A + Rev 1 primer and all reactions with Sample B + Rev 2 primer).

**Pause point:** You can store pooled PCR products at −20°C or proceed.

11. Run 5 μL of pooled PCR products and negative controls on a 1.5% TAE gel to confirm successful PCR. Expected band size should be approximately 350 bp.

***Note:*** This step can be performed in parallel with step 3 in purification of amplicons and gel quality control procedure.

***Note:*** Unused primers are usually visible on the gel at this stage (approximately 100 bp). These primers will be digested with Exonuclease I treatment during purification steps (see purification of amplicons and gel quality control test section).

***Note:*** A primer dimer band may appear on the gel in negative control lanes (approximately 200 bp). This is acceptable only if primer dimers are not present in reactions that receive DNA template.

12. Transfer half or less of your pooled reaction in a separate labeled microcentrifuge tube to process for purification. Store remainder of the unpurified products at −20°C.

***Note:*** The quantity of PCR products far exceeds the amount needed to submit for NGS. We recommend taking a fraction of this product to process for purification steps so that there is plenty of unpurified samples to work from in case errors are made during purification.

**Pause point:** You can store the aliquoted pooled PCR products at −20°C or proceed to purification steps.

**Table 2:** Forward PCR primer design

Primer	Stagger	Full primer sequence
NGS-Fwd-1	T	AATGATACGGCGACCACCGAGATCTACA CTCTTTCCCTACACGACGCTCTTCCGATCT**T** AAGTAGAGGCTTTATATATCTTGTGGAA AGGACGAAACACC
NGS-Fwd-2	AT	AATGATACGGCGACCACCGAGATCTACAC TCTTTCCCTACACGACGCTCTTCCGATCT**AT** CATGCTTAGCTTTATATATCTTGTGGAAAG GACGAAACACC
NGS-Fwd-3	GAT	AATGATACGGCGACCACCGAGATCT ACACTCTTTCCCTACACGACGCTCTTCCGATCT**GAT** GCACATCTGCTTTATATATCTTGTGGAAAG GACGAAACACC
NGS-Fwd-4	CGAT	AATGATACGGCGACCACCGAGATCTA CACTCTTTCCCTACACGACGCTCTTCCGATCT**CGAT** TGCTCGACGCTTTATATATCTTGTGGAA AGGACGAAACACC
NGS-Fwd-5	TCGAT	AATGATACGGCGACCACCGAGATC TACACTCTTTCCCTACACGACGCTCTTCCGATCT**TCGAT** AGCAATTCGCTTTATATATCTTGTGGAAA GGACGAAACACC
NGS-Fwd-6	ATCGAT	AATGATACGGCGACCACCGAGATCTACACT CTTTCCCTACACGACGCTCTTCCGATCT**ATCGAT** AGTTGCTTGCTTTATATATCTTGTGGAAAG GACGAAACACC
NGS-Fwd-7	GATCGAT	AATGATACGGCGACCACCGAGATCTAC ACTCTTTCCCTACACGACGCTCTTCCGATCT**GATCGAT** CCAGTTAGGCTTTATATATCTTGTGGAA AGGACGAAACACC
NGS-Fwd-8	CGATCGAT	AATGATACGGCGACCACCGAGATCTACA CTCTTTCCCTACACGACGCTCTTCCGATCT**CGATCGAT** TTGAGCCTGCTTTATATATCTTGTGGAAA GGACGAAACACC
NGS-Fwd-9	ACGATCGAT	AATGATACGGCGACCACCGAGATCTACACTC TTTCCCTACACGACGCTCTTCCGATCT**ACGATCGAT** ACACGATCGCTTTATATATCTTGTGGAAAGG ACGAAACACC
NGS-Fwd-10	TACGATCGAT	AATGATACGGCGACCACCGAGATCTACACTC TTTCCCTACACGACGCTCTTCCGATCT**TACGATCGAT** GGTCCAGAGCTTTATATATCTTGTGGAAAG GACGAAACACC

**Table 3:** Reverse PCR primer design

Primer	Barcode	Full primer sequence
KO-Rev-1	TCGCCTTG	CAAGCAGAAGACGGCATACGAGAT **TCGCCTTG**GTGACTGGAGTTCAGACGTGT GCTCTTCCGATCTAAGATCTAGTTACG CCAAGCTTAAA
KO-Rev-2	ATAGCGTC	CAAGCAGAAGACGGCATACGAGAT **ATAGCGTC**GTGACTGGAGTTCAGACGTGTGCT CTTCCGATCTAAGATCTAGTTACGCC AAGCTTAAA
KO-Rev-3	GAAGAAGT	CAAGCAGAAGACGGCATACGAGAT **GAAGAAGT**GTGACTGGAGTTCAGACGTGTGCTC TTCCGATCTAAGATCTAGTTACGCC AAGCTTAAA
KO-Rev-4	ATTCTAGG	CAAGCAGAAGACGGCATACGAGAT **ATTCTAGG**GTGACTGGAGTTCAGACGTGTGCTC TTCCGATCTAAGATCTAGTTACGC CAAGCTTAAA
KO-Rev-5	CGTTACCA	CAAGCAGAAGACGGCATACGAGAT **CGTTACCA**GTGACTGGAGTTCAGACGTGTGCTCT TCCGATCTAAGATCTAGTTACGCCAA GCTTAAA
KO-Rev-6	GTCTGATG	CAAGCAGAAGACGGCATACGAGAT **GTCTGATG**GTGACTGGAGTTCAGACGTGTG CTCTTCCGATCTAAGATCTAGTTACG CCAAGCTTAAA
KO-Rev-7	TTACGCAC	CAAGCAGAAGACGGCATACGAGAT **TTACGCAC**GTGACTGGAGTTCAGACGTGTG CTCTTCCGATCTAAGATCTAGTTACG CCAAGCTTAAA
KO-Rev-8	TTGAATAG	CAAGCAGAAGACGGCATACGAGAT **TTGAATAG**GTGACTGGAGTTCAGACGTGTGCT CTTCCGATCTAAGATCTAGTTACG CCAAGCTTAAA
KO-Rev-9	TATAGCCT	CAAGCAGAAGACGGCATACGAGAT **TATAGCCT**GTGACTGGAGTTCAGACGTGTGC TCTTCCGATCTAAGATCTAGTTAC GCCAAGCTTAAA
KO-Rev-10	ATAGAGGC	CAAGCAGAAGACGGCATACGAGAT **ATAGAGGC**GTGACTGGAGTTCAGACGTGTGC TCTTCCGATCTAAGATCTAGTTAC GCCAAGCTTAAA

### Purification of amplicons and gel quality control test

**Timing: 1–3 h**

The following procedure describes how to purify the PCR products before submission for NGS. Free single-stranded primers, which carry P5/P7 adapter sequences and can bind the Illumina flow cell during NGS, are removed through Exonuclease I digestion. Exonuclease I will degrade any single-stranded oligonucleotides, including free primers remaining after the PCR. The PCR products are then further purified to remove PCR components through spin-column purification using the GeneJET PCR Purification Kit.

13. Exonuclease I digest - Add 1 μL Exonuclease I for every 10 μL of reaction (i.e., Add 10 μL Exonuclease I for 100 μL of reaction).
14. Incubate reactions at 37°C for 1 h followed by a 20 min incubation at 80°C (see Table 6).

**Table 6:** Thermocycling conditions for Exonuclease I digestion

Steps	Temperature	Time	Cycles
Enzyme activation	37°C	60 min	1
Enzyme deactivation	80°C	20 min	1
Hold	4°C	infinite
15. Spin-column purify digested PCR products using the GeneJET PCR Purification Kit following manufacturer’s protocol (https://assets.fishersci.com/TFS-Assets/LSG/manuals/MAN0012662_GeneJET_PCR_Purification_UG.pdf). Elute in 50 μL Molecular Grade Water in the final step.

***Note:*** There is an optional step that recommends adding 100% isopropanol to increase yield of products ≤500 bp. Add 100% isopropanol as suggested in the manufacturer’s protocol (size of amplicon of interest is ∼350 bp).

**Figure 3:**
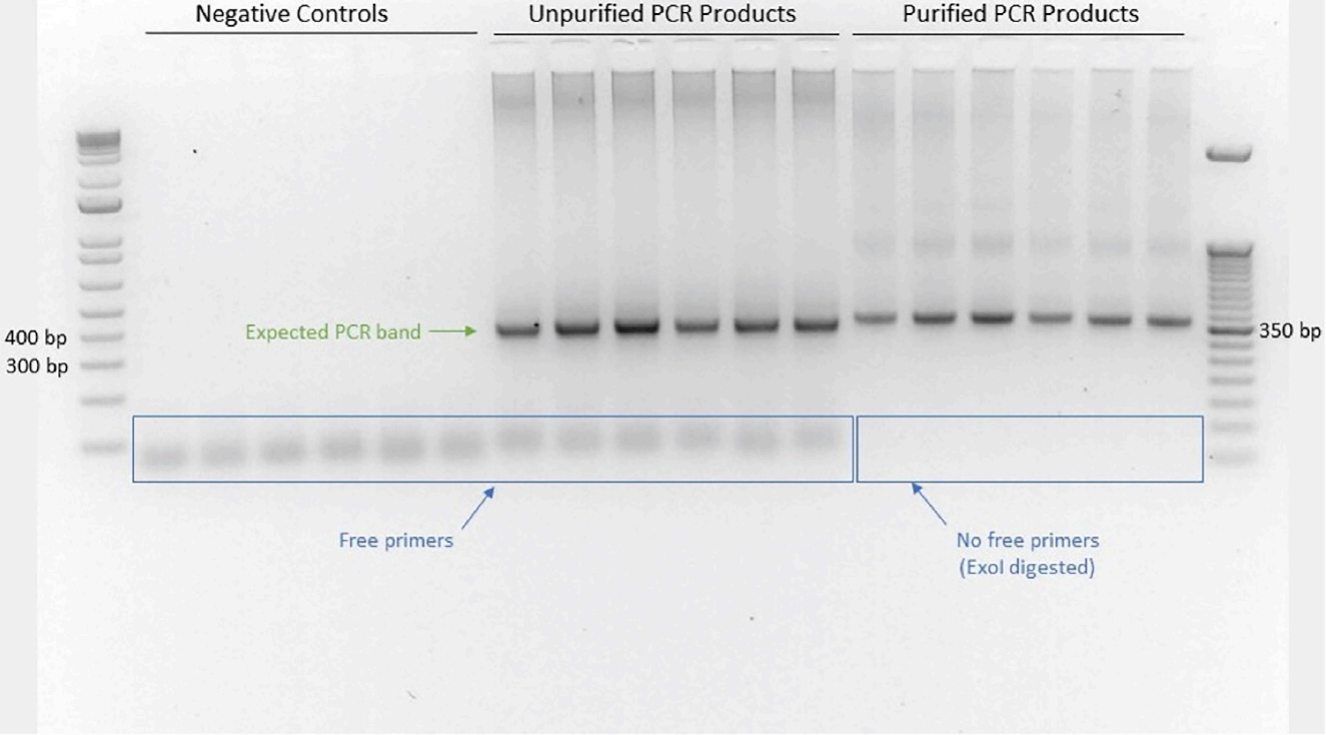
Gel quality control test of unpurified and purified PCR products One negative control for each independent sample (6 total) was run side by side with the unpurified and purified samples obtained in this protocol. Expected PCR band size is approximately 350 bp. Exonuclease I digest removes free primers from the PCR product. Negative control lanes should not have any visible bands other than free primers.

***Note:*** A faint band higher than the PCR product often appears on the gel after spin-column purification. This should not cause any sequencing problems (samples with this band have been submitted for NGS with no issues in quality). We speculate that the higher band is degraded gDNA that is carried over after purification. However, this DNA will not have the Illumina adapter sequences that were introduced via PCR and therefore will not influence NGS reads.

***Note:*** The expected band after purification may fall slightly higher than prior to purification on the agarose gel. This is likely due to slight changes in ionic concentration after purification affecting apparent molecular weight and should not provoke any concerns.

16. Prepare 5 μL of purified samples to run on a 1.5% Tris-Acetate-EDTA (TAE) gel. There should be no visible primers on the gel at this stage (see Figure 3).

17. Quantify the purified samples with the Qubit dsDNA HS Assay Kit using manufacturer’s protocol (https://assets.thermofisher.com/TFS-Assets/LSG/manuals/Qubit_dsDNA_HS_Assay_UG.pdf).

**Pause point:** Samples can be stored at −20°C for no more than 5 months until ready to submit for NGS.

## Expected outcomes

Figure 3 shows a representative gel with unpurified and purified PCR amplicons. If the purified samples pass this quality control step, they are ready to submit for NGS. Our lab uses Genewiz for NGS, but it is up to the researcher to choose an NGS provider. Typically, a provider of NGS services will perform one or more of the following quality control tests on samples: Tapestation, Qubit concentration reading, or quantitative PCR. This protocol produces 50 μL of purified sample and Genewiz requests 20 μL of purified sample to run quality control tests and to load onto the flow cell. Contact the NGS provider of your choice for details on sample submission requirements.

## Limitations

This protocol is only applicable and successful for cells that have been effectively transduced and selected for expression of the sgRNA library of interest. Cells that have not been transduced properly will not produce PCR products. This protocol is designed to be used for libraries containing the LentiGuide-Puro or LentiGuide-PuroV2 backbone, but may be adapted for use with other libraries by designing appropriate forward and reverse primers and calculating guide representation as outlined.

The gDNA yield represented in the calculations was established by our group in human cell lines but would need to be standardized separately in a different model system.

Variability in thermocycler efficiency may affect the product obtained using this protocol. This protocol was intended to be completed in a decontaminated PCR workstation. Performing this protocol on a standard benchtop may increase the likelihood of contamination and decrease the purity of the samples.

## Troubleshooting

### Problem 1

Low gDNA yield during gDNA extraction (step 3 in gDNA extraction).

### Potential solution

Perform a second elution step to recover more gDNA from spin column (see “Optional” step in step 2 of gDNA extraction). Potential causes of low gDNA yields include clogged filters from processing too many cells or ethanol contamination during washing steps. Ensure that there is no remaining wash buffer above spin column after washes. You may perform an additional 1-min spin centrifuge at maximum speed prior to eluting in water to remove any residual wash buffer.

### Problem 2

Concentration of gDNA is <190 ng/μL and 4 μg of template cannot fit a 50 μL PCR reaction set up (step 3 in one-step PCR sample preparation for NGS).

### Potential solution

You may input 2 μg of template instead of 4 μg per reaction. However, you will need to double the number of PCR reactions per sample to capture the same library coverage.

### Problem 3

PCR fails and there is no visible band of expected size on the agarose gel (step 8 in one-step PCR sample preparation for NGS).

### Potential solution

Ethanol contamination in gDNA solution can inhibit the PCR reaction. One indication of ethanol contamination is if the gDNA in solution does not freeze at −20°C. gDNA in pure water will freeze at this temperature. You may repeat PCR reaction using 2 μg input template instead of 4 μg to dilute the contaminating ethanol in the PCR set up and reduce its inhibitory effects in the reaction.

### Problem 4

Free primers remain in the purified PCR products and is visible on the agarose gel (step 4 in purification of amplicons and gel quality control test).

### Potential solution

You may repeat Exonuclease I digestion and re-purify samples using spin-column purification.

### Problem 5

Unexpected PCR products in negative control lanes.

### Potential solution

PCR components, pipettes, or consumables are contaminated with plasmid or PCR products. Make fresh aliquots of reagents and decontaminate work areas.

## Resource availability

### Lead contact

Further information and requests for resources and reagents should be directed to and will be fulfilled by the lead contact, Charles M. Rudin (rudinc@mskcc.org).

### Materials availability

Unique reverse barcoding primers were adapted for use in this protocol. These primers can be requested by contacting the lead contact, Charles M. Rudin (refer to the primer sequence Table 3). The Saturn V library referenced in this protocol can be requested by contacting the lead contact, Charles M. Rudin.

### Data and code availability

This study did not generate datasets or code.
